# An elevated C-reactive protein concentration, prior to surgery, predicts poor cancer-specific survival in patients undergoing resection for gastro-oesophageal cancer

**DOI:** 10.1038/sj.bjc.6603150

**Published:** 2006-05-09

**Authors:** A B C Crumley, D C McMillan, M McKernan, J J Going, C J Shearer, R C Stuart

**Affiliations:** 1 University Department of Surgery, Royal Infirmary, Glasgow G31 2ER, UK; 2 University Department of Pathology, Royal Infirmary, Glasgow G31 2ER, UK

**Keywords:** gastro-oesophageal cancer, resection, TNM stage, C-reactive protein, survival

## Abstract

There is increasing evidence that the presence of an ongoing systemic inflammatory response is associated with poor outcome in patients undergoing resection for a variety of tumours. The aim of the present study was to examine the relationship between clinico-pathological status, preoperative C-reactive protein concentration and cancer-specific survival in patients undergoing resection for gastro-oesophageal cancer. One hundred and twenty patients attending the upper gastrointestinal surgical unit in the Royal Infirmary, Glasgow, who were selected for potentially curative surgery, were included in the study. Laboratory measurements of haemoglobin, white cell, lymphocyte and platelet counts, albumin and C-reactive protein were carried out at the time of diagnosis. All patients underwent en-bloc resection with lymphadenectomy and survived at least 30 days following surgery. On multivariate analysis, only the positive to total lymph node ratio (hazard ratio (HR) 2.02, 95% confidence interval (CI) 1.44–2.84, *P*<0.001) and preoperative C-reactive protein concentration (HR 3.53, 95% CI 1.88–6.64, *P*<0.001) were independent predictors of cancer-specific survival. The patient group with no evidence of a preoperative systemic inflammatory response (C-reactive protein ⩽10 mg l^−1^) had a median survival of 79 months compared with 19 months in the elevated systemic inflammatory response group (*P*<0.001). The results of the present study indicate that in patients selected to undergo potentially curative resection for gastro-oesophageal cancer, the presence of an elevated preoperative C-reactive protein concentration is an independent predictor of poor cancer-specific survival.

Gastro-oesophageal cancer is the third commonest cause of cancer death in the UK. Each year, there are approximately 16 500 new cases and over 13 000 deaths attributable to the disease. Overall survival is poor with the majority of patients presenting with advanced, inoperable disease and less than 15% surviving 5 years (Cancerstats, 2004; www.cancerresearchuk.org). Surgery confers the greatest chance of long-term cure but is associated with appreciable morbidity and mortality. As a consequence, potentially curative surgery is carried out relatively infrequently.

The prognosis for patients who undergo potentially curative resection is influenced by various pathologic characteristics of the resected tumour specimen. In particular, residual tumour (R), lymph node status and the ratio of positive to total lymph nodes sampled have been shown to have independent prognostic value (Roder *et al*, 1994; Siewert *et al*, 1998).

It is increasingly recognised that it is not only the intrinsic properties of tumour cells that determine tumour spread but also the host inflammatory response (Balkwill and Mantovani, 2001; Coussens and Werb, 2002). Indeed, the systemic inflammatory response, as evidenced by elevated circulating concentrations of C-reactive protein, has been shown to be a disease-independent prognostic factor in a variety of tumours, when resections are carried out with curative intent (McMillan *et al*, 2003; Hilmy *et al*, 2005; Jamieson *et al*, 2005).

An elevated serum C-reactive protein concentration, before surgery, has previously been shown to have independent prognostic value in patients with resectable oesophageal cancer (Nozoe *et al*, 2001; Ikeda *et al*, 2003; Shimada *et al*, 2003). However, some of these studies included patients with metastatic disease at the time of surgery and used variable C-reactive protein thresholds. To date, the prognostic value of C-reactive protein has not been examined in patients undergoing potentially curative resection for gastric cancer.

The aim of the present study was to examine the relationship between clinico-pathological status, C-reactive protein concentration, measured before surgery, and cancer-specific survival in patients selected for potentially curative resection of gastro-oesophageal cancer.

## PATIENTS AND METHODS

### Patients

Patients selected for potentially curative resection of gastro-oesophageal cancer (between January 1996 and December 2004) and who had a preoperative measurement of C-reactive protein were included in the study. For gastric cancers, tumour node metastasis (TNM) stage I–III tumours were considered to be amenable to curative surgical resection. For oesophageal cancers, TNM stage I–III tumours, excluding T4, were considered to be amenable to curative surgical resection. Measurements of haemoglobin, white cell, lymphocyte and platelet counts, albumin and C-reactive protein were carried out before staging laparoscopy or surgery. All patients underwent en-bloc resection with lymphadenectomy (median 20, range 3–55 nodes resected). All patients were treated in the upper gastrointestinal (GI) surgical unit at Glasgow Royal Infirmary and survived at least 30 days following surgery. Patients undergoing neo-adjuvant chemotherapy or radiotherapy were excluded.

Data for 1996–1998 (*n*=16) were collected retrospectively and that for 1999–2004 (*n*=104) prospectively.

The study was approved by the Research Ethics Committee of Glasgow Royal Infirmary.

### Methods

The extent of tumour spread was recorded using the TNM stage. Tumours of the gastro-oesophageal junction were further classified according to site, using the Siewert system; type 1 and 2 lesions of the gastro-oesophageal junction were designated, as cancers of the oesophagus. Type 3 tumours of the cardia were designated gastric cancers.

Routine preoperative laboratory measurements of haemoglobin, white cell, lymphocyte and platelet counts, albumin and C-reactive protein were carried out. The coefficient of variation for these methods, over the range of measurement, was less than 10% as established by routine quality control procedures. The limit of detection of the assay is a C-reactive protein concentration of less than 6 mg l^−1^, with the upper limit of normal values being ⩽10 mg l^−1^. Based on previous work (O'Gorman *et al*, 2000; McMillan *et al*, 2001), a C-reactive protein concentration of greater than 10 mg l^−1^ was considered to indicate the presence of a systemic inflammatory response.

### Statistics

Data are presented as median and range. Comparisons between groups of patients were carried out using contingency table analysis (*χ*^2^) as appropriate. Grouping of the laboratory variables haemoglobin, white cell, lymphocyte and platelet counts, albumin and C-reactive protein was carried out using standard thresholds (O'Gorman *et al*, 2000; McMillan *et al*, 2001; Ikeda *et al*, 2002; Maltoni *et al*, 2005; Shen *et al*, 2005). Survival (cancer-specific) analysis of the group variables was performed using the Cox proportional hazard model. Deaths up to the end of February 2006 have been included in the analysis. Multivariate survival analysis, including all covariates that were significant on univariate analysis, was performed using a stepwise backward procedure to derive a final model of the variables that had a significant independent relationship with survival. To remove a variable from the model, the corresponding *P*-value had to be greater than 0.10. Analysis was performed using SPSS software (SPSS Inc., Chicago, IL, USA).

## RESULTS

Baseline clinico-pathological characteristics of the patients (*n*=120) studied are shown in Table 1. The majority of patients were male, under 65 years and had adenocarcinoma. The majority of patients had localised disease with clear resection margins and therefore underwent potentially curative surgery (*n*=99). Of the remaining 21 patients, three patients were found to have metastasis at the time of surgery; one patient with a small bowel deposit, one patient with a single liver deposit and one patient with metastatic disease affecting the terminal ileum/ascending colon. The remaining 18 patients had positive circumferential resection margins of the oesophagus (tumour <1 mm from resection margin).

The majority of patients had laboratory-based measures including haemoglobin, white cell, lymphocyte and platelet counts, albumin and C-reactive protein concentrations in the normal range. Fifteen patients (13%) had an elevated circulating C-reactive protein concentration (>10 mg l^−1^) before surgery.

**Figure 1:**
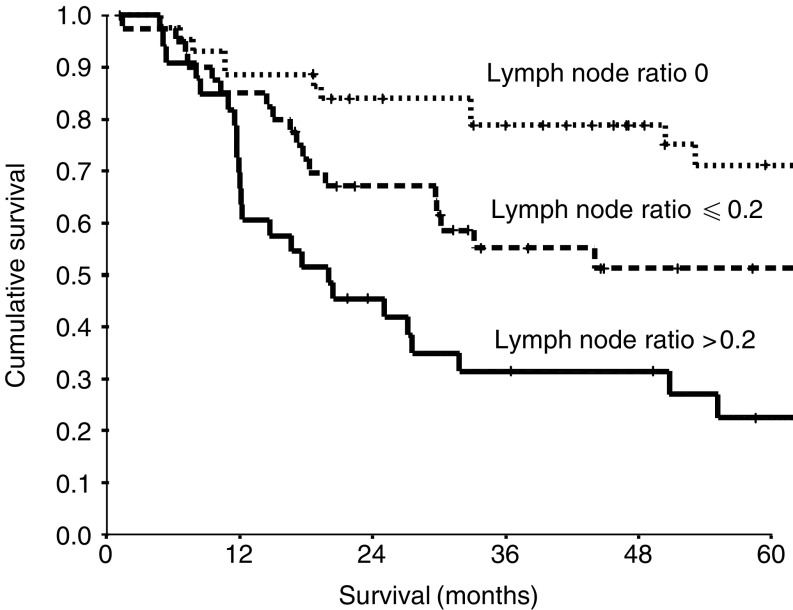
The relationship between the positive to total lymph node ratio and cancer-specific survival in patients undergoing resection for gastro-oesophageal cancer.

**Figure 2:**
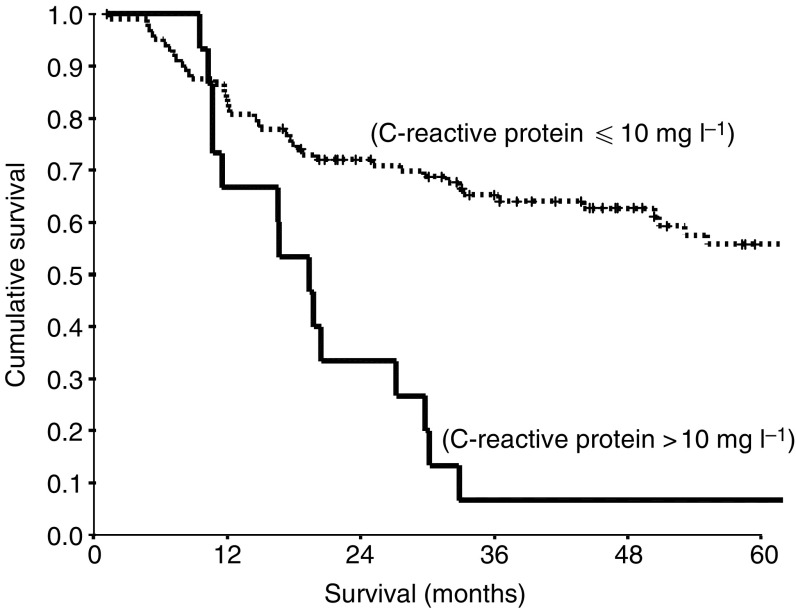
The relationship between the systemic inflammatory response, as evidenced by C-reactive protein concentrations, and cancer-specific survival in patients undergoing resection for gastro-oesophageal cancer.

During the follow-up period, 60 (50%) patients died; 58 of their disease. The median follow-up of the survivors was 55 months. On univariate analysis, pTNM stage (*P*<0.01), lymph node status (*P*<0.01), positive to total lymph node ratio (*P*<0.001) and preoperative C-reactive protein (*P*<0.001) were significantly associated with survival (Table 1). On multivariate analysis of these significant variables, positive to total lymph node ratio (hazard ratio (HR) 2.02, 95% confidence interval (CI) 1.44–2.84, *P*<0.001, Figure 1) and preoperative C-reactive protein (HR 3.53, 95% CI 1.88–6.64, *P*<0.001, Figure 2) retained independent significance.

The preoperative values of C-reactive protein at the thresholds of >5 and >10 mg l^−1^ were compared in multivariate survival analysis. In this analysis, the prognostic significance of the threshold of >10 mg l^−1^ (*P*=0.013) was greater than >5 mg l^−1^ (*P*=0.559).

The relationship between the presence of an elevated preoperative C-reactive protein concentration and clinico-pathological characteristics is shown in Table 2. There was no significant difference in age, sex, tumour site, tumour type, pTNM stage, presence of positive resection margins, lymph node status, positive to total lymph node ratio, haemoglobin, white cell, lymphocyte and platelet counts and albumin groupings between the inflammatory and non-inflammatory groups. In contrast, a greater proportion of patients had a lower haemoglobin (*P*<0.05) and percentage lymphocyte counts (*P*<0.001) in the elevated systemic inflammatory response group.

The patient group with no evidence of a preoperative systemic inflammatory response (C-reactive protein ⩽10 mg l^−1^) had a median survival of 79 months compared with 19 months in the elevated systemic inflammatory response group (*P*<0.001). The 1- and 2-year survival rates in the patient group with no evidence of a preoperative systemic inflammatory response were 83 and 72%, respectively, compared with 67 and 33%, respectively, in the elevated systemic inflammatory response group.

## DISCUSSION

Surgical resection remains the best prospect for long-term survival in patients with gastro-oesophageal cancer. Currently, in patients undergoing surgery, prognostic factors are based on the pathological findings from the resected tumour. However, this means that the assessment of prognosis occurs after a major operation with significant morbidity and mortality. Therefore, it is of interest that in the present study, an elevated circulating concentration of C-reactive protein (>10 mg l^−1^), measured preoperatively, was associated with poor survival, independent of the pathological positive to total lymph node ratio or pTNM stage. In contrast, neither anaemia, leucocytosis, lymphocytopenia or thrombocytosis predicted survival in this group of patients undergoing resection for gastro-oesophageal cancer.

There have been three previous studies from Japan that have shown the prognostic value of an elevated C-reactive protein concentration in patients undergoing resection for oesophageal cancer. However, in contrast with the present study, they included patients with primarily (>90%) squamous tumours (Nozoe *et al*, 2001; Ikeda *et al*, 2003; Shimada *et al*, 2003), patients receiving neo-adjuvant treatment (Nozoe *et al*, 2001; Ikeda *et al*, 2003), patients who had advanced disease before surgery (Nozoe *et al*, 2001; Ikeda *et al*, 2003), they used a threshold for C-reactive protein of >5 mg l^−1^ (Nozoe *et al*, 2001; Ikeda *et al*, 2003) and the positive to total lymph node ratio was not assessed (Nozoe *et al*, 2001; Ikeda *et al*, 2003; Shimada *et al*, 2003).

In contrast to the above studies, the majority of patients in the present study had adenocarcinoma, reflecting the prevailing type in the Western world. Also, only those patients who underwent potentially curative resection and did not receive neo-adjuvant treatment were included in the present study. In these patients, the positive to total lymph node ratio was prognostic independent of an elevated C-reactive protein concentration, before surgery.

Therefore, the results of the present study are consistent with C-reactive protein, measured before surgery, having prognostic value independent of established pathological criteria in patients with resectable oesophageal cancer (Nozoe *et al*, 2001; Ikeda *et al*, 2003; Shimada *et al*, 2003). Moreover, we have shown that an elevated C-reactive protein concentration, before surgery, is associated with poor cancer-specific survival in patients undergoing potentially curative resection for gastro-oesophageal adenocarcinoma.

It was of interest that the threshold for C-reactive protein (>10 mg l^−1^), which we established in previous studies in patients with gastro-intestinal cancer (O'Gorman *et al*, 2000; McMillan *et al*, 2001, 2003; Crumley *et al*, 2006), was superior to that (>5 mg l^−1^) used in previous prognostic studies in oesophageal cancer (Nozoe *et al*, 2001; Ikeda *et al*, 2003). Moreover, compared with patients undergoing potentially curative resection for colorectal cancer in the same institution (McMillan *et al*, 2003), the proportion of patients who had an elevated circulating concentration of C-reactive protein (>10 mg l^−1^) preoperatively was lower in the present study (13 *vs* 28%). This probably reflects, given the increased morbidity and mortality associated with gastro-oesophageal surgery, a more selective approach than that in colorectal cancer. Nevertheless, taken together, these results show the utility of C-reactive protein in the preoperative assessment of patients undergoing potentially curative surgery for GI cancer. Indeed, the combination of pathological stage and C-reactive protein has recently been used to improve the prediction of outcome in patients who underwent potentially curative resection for colorectal cancer (Canna *et al*, 2004).

The basis of the independent relationship between an elevated C-reactive protein concentration and poor survival in gastro-oesophageal cancer is not clear. There are a number of possible explanations. Firstly, that an elevated C-reactive protein identifies those patients with an impaired T-lymphocytic response, as poor infiltration of GI tumours appears to be associated with poor outcome (Schumacher *et al*, 2001; Ali *et al*, 2004) and an elevated C-reactive protein concentration has recently been shown to be inversely associated with T-lymphocyte subset infiltration (Canna *et al*, 2005). Indeed, in the present study, an elevated C-reactive protein concentration was associated with greater proportion of patients having lymphocytopenia.

An alternative explanation is that an elevated C-reactive protein concentration may identify those patients with a proangiogenic environment, as angiogenesis is associated with poor outcome in patients with GI tumours (Tanigawa *et al*, 1997; Fondevila *et al*, 2004) and circulating concentrations of vascular endothelial growth factor are directly associated with C-reactive protein (Xavier *et al*, 2006). Clearly, both these mechanisms may be related and promote unrestrained tumour growth and the dissemination required for the greater malignant potential associated with an elevated C-reactive protein concentration.

This is a relatively small study in a single centre and requires verification in large cohorts in other centres. If an elevated C-reactive protein concentration is confirmed to predict a poorer prognosis, it may be the case that patients staged to have potentially resectable gastro-oesophageal cancer, yet a high inflammatory profile preoperatively, should not undergo surgery. Alternatively, modulation of the systemic inflammatory response may be a useful approach in these patients in the preoperative period.

In summary, the results of the present study indicate that in patients selected to undergo potentially curative resection for gastro-oesophageal cancer, the presence of an elevated C-reactive protein concentration preoperatively (>10 mg l^−1^) is an independent predictor of poor cancer-specific survival.

## Figures and Tables

**Table 1:** Clinico-pathological characteristics of patients selected for potentially curative resection for gastro-oesophageal cancer: univariate survival analysis

	**Patients (*n*=120)**	**HR (95% CI)**	**(*P*-value)**
Age (⩽65/65–74/⩾75 years)	60/47/13	1.34 (0.92–1.95)	0.132
Sex (m/f)	80/40	1.18 (0.69–2.04)	0.543
Tumour site (oesophageal/gastric)	60/60	1.28 (0.76–2.14)	0.358
Tumour type (adenocarcinoma/squamous)	100/20	1.24 (0.63–2.47)	0.530
pTNM stage (I/II/III/IV)	32/35/49/4	1.59 (1.15–2.21)	0.006
Resection margin R0/R1	99/19	1.49 (0.79–2.83)	0.218
Lymph node status (−/+)	44/74	2.84 (1.49–5.41)	0.002
Positive to total lymph node ratio (0/⩽0.2/>0.2)	44/41/33	2.03 (1.45–2.86)	<0.001
Haemoglobin (⩾12/<12 g l^−1^)	95/23	1.42 (0.76–2.65)	0.267
White cell count (<8.5/8.5–11.0/>11.0 ( × 10^9^ l^−1^))	79/30/9	1.05 (0.70–1.58)	0.800
Lymphocyte percentage (20–40/12–19.9/0–11.9%)	97/17/4	1.38 (0.83–2.30)	0.211
Platelets (<400/⩾400 ( × 10^9^ l^−1^))	110/7	0.69 (0.22–2.23)	0.541
Albumin (⩾35/<35 g l^−1^)	117/2	0.96 (0.13–7.00)	0.970
C-reactive protein (⩽10/>10 mg l^−1^)	105/15	3.51 (1.89–6.53)	<0.001

CI=confidence interval; HR=hazard ratio.

**Table 2:** The relationship between the presence of a preoperative systemic inflammatory response and clinico-pathological characteristics of gastro-oesophageal cancer

	**C-reactive protein ⩽10 mg l^−1^ (*n*=105)**	**C-reactive protein >10 mg l^−1^ (*n*=15)**	**(*P*-value)**
Age (⩽65/65–74/⩾75 years)	54/39/12	6/8/1	0.721
Sex (m/f)	72/33	8/7	0.244
Tumour site (oesophageal/gastric)	54/51	6/9	0.410
Tumour type (adenocarcinoma/squamous)	88/17	12/3	0.712
pTNM stage (I/II/III/IV)	31/29/41/4	1/6/8/0	0.223
Resection margin R0/R1	88/15	11/4	0.235
Lymph node status (−/+)	39/64	5/10	0.736
Positive to total lymph node ratio (0/⩽0.2/>0.2)	39/35/29	5/6/4	0.891
Haemoglobin (⩾12/<12 g l^−1^)	86/17	9/6	0.033
White cell count (<8.5/8.5–11.0/>11.0 ( × 10^9^ l^−1^))	71/25/7	8/5/2	0.204
Lymphocyte percentage (20–40/12–19.9/0–11.9%)	90/11/2	7/6/2	<0.001
Platelets (<400/⩾400 ( × 10^9^ l^−1^))	96/6	14/1	0.905
Albumin (⩾35/<35 g l^−1^)	102/2	15/0	0.590
Survival (months)^a^	79.2 (53.8–104.6)	19.4 (15.3–23.4)	<0.001

CI=confidence interval.

a Median (95% CI).
